# Pasteurella multocida Bacteremia in a Young Infant

**DOI:** 10.7759/cureus.99559

**Published:** 2025-12-18

**Authors:** Ariana Goncalves Marques, Elsa Guimarães, Margarida Pereira, João Agro

**Affiliations:** 1 Pediatrics Department, Unidade Local de Saúde da Região de Leiria, Leiria, PRT

**Keywords:** bacteremia, febrile infant, high clinical suspicion, household pets, pasteurella multocida

## Abstract

*Pasteurella multocida *is a rare cause of infection in the pediatric age group that can lead to severe systemic disease and is associated with significant morbidity and mortality. This bacterium is found in the normal nasopharyngeal flora of domestic animals. Transmission can occur through direct traumatic contact or indirectly via animal saliva. We present the case of a 30-day-old male infant who attended a neonatology follow-up visit with no parental concerns. On physical examination, the infant showed intermittent periods of whining, ill appearance, reduced vitality, and poor peripheral perfusion. There was no history of fever. No relevant epidemiological context was identified. He had contact with a dog at home. Blood tests were performed, revealing hyperglycemia (256 mg/dL) and a slight increase in C-reactive protein (18.3 mg/L), with normal blood count and lactate levels. After hospital admission, he developed fever and molting skin. Subsequent blood tests revealed increased C-reactive protein (75.5 mg/L) and slightly raised lactate (3.5 mmol/L), with persistent hyperglycemia (181 mg/dL). He began empirical antibiotic therapy with ceftriaxone and ampicillin. Blood culture was positive for *P. multocida*, susceptible to all tested antibiotics; therefore, ceftriaxone was suspended, and ampicillin was continued for 14 days. He had a favorable clinical course. After discharge, follow-up visits were maintained until two years of age, and no abnormalities in growth or development were identified. This case aims to highlight that indirect contact through respiratory droplets or licking from domestic animals may transmit the infection. Therefore, it is important to remind the parents of the risks associated with close contact between animals and infants and advise them on hygienic measures when handling their children.

## Introduction

*Pasteurella multocida* is a facultative anaerobic, Gram-negative coccobacillus, found in the normal nasopharyngeal flora of 70-90% of cats and 20-50% of dogs. It can also be isolated from domestic cattle, rabbits, pigs, birds, and a wide range of wild species [1-5]. Human carriage of *P. multocida* is rare, but commensal colonization may occur following frequent animal contact [4].

In infants, *P. multocida* infection is a rare but serious bacterial disease associated with significant morbidity and mortality [1,2]. *P. multocida* usually causes soft-tissue infections with localized abscesses and lymphadenitis, but, in infants, it may lead to severe systemic disease, including meningitis, septicemia, pneumonia, conjunctivitis, and osteoarticular infection [1,3,4]. Although infection is usually associated with traumatic contact with domestic animals, through bites or scratches, most cases in this age group do not report prior traumatic contact [1,2]. Infection is thought to be acquired through indirect contact with animal saliva (licking) via family members, who may subsequently transmit the organism to the infant during care [1,3,6]. Vertical transmission has also been reported as a possible route of infection in this age group [3,7]. The present report highlights the potential risk of *P. multocida* infection in infants, even in the absence of traumatic contact with domestic animals.

This article was previously presented as a meeting abstract at the 41st Annual Meeting of the European Society for Paediatric Infectious Diseases in May 2023.

## Case presentation

A 30-day-old male infant presented to a neonatology follow-up with no parental concerns. Nevertheless, physical examination revealed intermittent periods of whining, reduced vitality, and poor peripheral perfusion, with no other abnormal findings. There was no history of fever, prostration, respiratory or gastrointestinal symptoms, or rash. He showed no feeding difficulties. No relevant epidemiological context was identified. He had household exposure to a dog.

The infant was born following an uneventful, well-monitored pregnancy. First-trimester maternal serology indicated primary syphilis, treated with penicillin; the remaining serologies were normal. Prenatal ultrasounds revealed no significant abnormalities. He was delivered at 37 weeks, with an Apgar score of 10/10/10. Neonatal screenings were unremarkable. Laboratory assessment at birth showed a negative non-treponemal test.

Blood tests were performed, revealing hyperglycemia and a slight increase in C-reactive protein. The laboratory results, along with their corresponding reference value, are presented in Table 1. Blood count showed no relevant changes, and lactate was normal. A urine dipstick test was performed, with no abnormalities. Blood and urine cultures were collected.

**Table 1:** Laboratory results at the neonatology follow-up visit and six hours after admission with reference ranges.

Parameter	At the neonatology follow-up visit	Six hours after admission	Reference value
Leucocytes (10^9^/L)	10.3	12.7	6.0–18.0
Neutrophils (10^9^/L)	9.0	9.1	1.2–7.5
Hemoglobin (g/dL)	12.0	11.8	9.0–14.0
Platelets (10^9^/L)	272.0	343.0	200.0–550.0
Glycemia (mg/dL)	256	181	50.0–80.0
C-reactive protein (mg/L)	18.3	75.5	5.0
Lactate (mmol/L)	2	3.5	5

He was admitted to the hospital. Within six hours of admission, he developed fever, whining, and mottled skin (cutis marmorata). Subsequent blood tests demonstrated increased C-reactive protein and slightly raised lactate, with persistent hyperglycemia (Table 1). A second blood culture was done. Although a lumbar puncture was performed, it was traumatic, and the cerebrospinal fluid (CSF) obtained was insufficient for cell count or biochemical analysis, being used only for culture.

Empiric antibiotic therapy with ceftriaxone and ampicillin was initiated. He had a favorable clinical course, with sustained apyrexia after 48 hours of antibiotic therapy and no further episodes of hyperglycemia. He remained hemodynamically stable, breathing spontaneously without need for supplemental oxygen, with good urine output and normal feeding. The first hemoculture was positive for *P.multocida*, susceptible to all tested antibiotics. Ceftriaxone was therefore suspended, with ampicillin continued for 14 days. CSF and urine cultures were negative.

After the antibiotic course, the patient was discharged and continued follow-up in the Neonatology appointment until two years of age, with normal growth and development.

## Discussion

*P. multocida* may cause severe systemic disease in infants, most commonly presenting as meningitis, followed by septicemia. Wei et al. reviewed 492 cases of *P. multocida* infection, including 70 in children. Among children, central nervous system infections were the most frequent, with meningitis reported mainly in infants younger than two months. Less frequently, cases of sepsis and respiratory infections were reported [8]. Nakwan et al. reported a review of 25 infants with *P. multocida* infection. The most frequent clinical presentation was bacteremia with meningitis, followed by isolated bacteremia and isolated meningitis. Less common manifestations included pneumonia, osteomyelitis, and conjunctivitis [3]. The present report describes a case of *P. multocida* bacteremia in a 30-day-old infant. The detection of subtle abnormalities on physical examination at the neonatology follow-up visit, i.e., intermittent whining, reduced vitality, and poor peripheral perfusion, prompted an immediate laboratory assessment, including blood culture. Marked hyperglycemia (256 mg/dL) and a mildly raised C-reactive protein led to hospital admission. Within six hours, he developed fever, persistent whining, and mottled skin, accompanied by a further rise in C-reactive protein and a slight increase in lactate. Urine and CSF cultures were performed as part of the sepsis evaluation. Empiric antibiotic therapy was initiated, and the blood culture confirmed *P. multocida* bacteremia. The only epidemiological factor indicative of this pathogen was the history of close contact with a household dog.

Infections are most commonly acquired through scratches and bite wounds from domestic animals [3,4,8,9]. Recent evidence indicates that indirect exposure can also contribute to infection [1,3,8]. Nakwan et al. reported that approximately half of all cases had a history of exposure to domestic animals, with non-traumatic exposure being more frequent than traumatic exposure [3]. On the other hand, Inglis et al. reviewed 74 cases of *P. multocida* infection in infants under one year of age and reported that most occurred without direct household animal contact [1]. In our case, the infant had contact with a domestic dog, but no traumatic exposure was reported. Different mechanisms have been suggested for the transmission of *P. multocida* infection in the absence of a bite or scratch [3,6,10]. One possibility is the contact with animal saliva through respiratory droplets or licking [3,9]. Indirect transmission through the hands of a family member is another possible route [6,9,10]. Therefore, prevention of *P. multocida* infections in infants relies on simple hygienic measures, such as hand washing, to minimize the risk of contact with the saliva of dogs or cats [6,10].

First-line treatment for *P. multocida* infection is penicillin or ampicillin [1,2,9]. Although rare, resistance to these antibiotics has been reported, usually mediated by β-lactamases such as ROB-1 or TEM-1, highlighting the need for antibiotic susceptibility testing. Treatment can be adjusted according to the antibiotic susceptibility results [9,11]. Treatment is recommended for 7-10 days in local infections and two weeks in neonatal bacteremia and meningitis [2,3,9].

Infection with *P. multocida* in infants is rare but is associated with significant morbidity and mortality. Nakwan et al. reported a mortality rate of 20% in neonatal infection [3]. Wade et al. described a lower mortality rate of 0.9% in *P. multocida* meningitis, although neurological complications were common, including seizure disorders, hemiparesis, and hydrocephalus [6]. In the absence of neurological complications during the acute phase of *P. multocida* meningitis, the prognosis is typically good, with normal psychomotor development [9]. In the present case, the infant had a good outcome. Only bacteremia was confirmed, which is associated with a better prognosis. Even if meningitis had been established, the absence of neurological complications would have suggested a mild disease course and a good overall outcome.

In this case, the combination of subtle clinical findings and the unexpected detection of hyperglycemia supported early suspicion of infection. In infants, hyperglycemia may occur in the context of infection through the stress response, reduced insulin secretion, and diminished peripheral glucose utilization [12]. Close clinical reassessment is essential to detect rapid changes in the infant’s condition. A complete sepsis screen, including blood, urine, and CSF cultures when feasible, is crucial once infection is suspected. Empiric antibiotic therapy should be initiated promptly and subsequently refined according to microbiological results. Finally, incorporating epidemiological context, particularly exposure to domestic animals, even without trauma, may help identify the risk factors for rare pathogens such as *P. multocida*. Early recognition and timely management are key, as they significantly improve clinical outcomes in these rare but potentially severe infections.

## Conclusions

Domestic animals, such as dogs and cats, are common family members. Direct contact with these animals and inadequate hand hygiene increases the risk of *P. multocida* infection, which can lead, especially during the neonatal period, to severe and potentially life-threatening complications. Parents should be reminded of the risks associated with close contact between animals and infants and advised on preventive measures such as avoiding the pet’s access to the infant’s bedroom, preventing the dog from licking the infant, and thoroughly washing their hands after handling their pets. This case report also highlights the importance of early clinical suspicion and careful interpretation of hyperglycemia in laboratory findings to recognize early presentations of *P. multocida* infection, as most reported infant cases occurred without direct trauma.

## References

[1] Inglis C, Wen SC, Kapoor V (2025). Infantile Pasteurella multocida meningitis: case report and review of the literature. Pediatr Infect Dis J.

[2] Yamaguchi H, Tamura T, Abe M (2014). Prolonged incubation period in neonatal Pasteurella multocida meningitis and bacteremia. Pediatr Int.

[3] Nakwan N, Nakwan N, Atta T, Chokephaibulkit K (2009). Neonatal pasteurellosis: a review of reported cases. Arch Dis Child Fetal Neonatal Ed.

[4] Edwards MS (2025). Pasteurella multocida. Rudolph's Pediatrics, 22e.

[5] Mogilner L, Katz C (2019). Pasteurella multocida. Pediatr Rev.

[6] Wade T, Booy R, Teare EL, Kroll S (1999). Pasteurella multocida meningitis in infancy - (a lick may be as bad as a bite). Eur J Pediatr.

[7] Zaramella P, Zamorani E, Freato F, Cattai M, Meloni GA (1999). Neonatal meningitis due to a vertical transmission of Pasteurella multocida. Pediatr Int.

[8] Wei B, Liu C, Zhu J, Zou X, Zhang Z (2025). Pasteurella multocida infection: a differential retrospective study of 482 cases of P. multocida infection in patient of different ages. BMC Infect Dis.

[9] Guet-Revillet H, Levy C, Andriantahina I (2013). Paediatric epidemiology of Pasteurella multocida meningitis in France and review of the literature. Eur J Clin Microbiol Infect Dis.

[10] Cohen-Adam D, Marcus N, Scheuerman O, Hoffer V, Garty BZ (2006). Pasteurella multocida septicemia in a newborn without scratches, licks or bites. Isr Med Assoc J.

[11] Ni MM, Lin WX, Li Y, Qiu JC, Wu CF (2025). Rare infantile sepsis and meningitis caused by Pasteurella multocida: a case report. J Infect Public Health.

[12] Mittal A, Gupta R, Sharma S, Aggarwal KC (2013). Stress induced hyperglycemia in a term baby mimicking diabetic ketoacidosis with stroke. J Clin Neonatol.

